# Dramatically Enhanced Visible Light Response of Monolayer ZrS_2_ via Non-covalent Modification by Double-Ring Tubular B_20_ Cluster

**DOI:** 10.1186/s11671-016-1719-8

**Published:** 2016-11-10

**Authors:** Yuan Si, Hong-Yu Wu, Hao-Ming Yang, Wei-Qing Huang, Ke Yang, Ping Peng, Gui-Fang Huang

**Affiliations:** 1 Department of Applied Physics, School of Physics and Electronics, Hunan University, Changsha, 410082 China; 2 School of Materials Science and Engineering, Hunan University, Changsha, 410082 China

**Keywords:** Electronic structure, Enhanced visible-light response, ZrS_2_/B_20_ hybrid, First-principles

## Abstract

The ability to strongly absorb light is central to solar energy conversion. We demonstrate here that the hybrid of monolayer ZrS_2_ and double-ring tubular B_20_ cluster exhibits dramatically enhanced light absorption in the entire visible spectrum. The unique near-gap electronic structure and large built-in potential at the interface will lead to the robust separation of photoexcited charge carriers in the hybrid. Interestingly, some Zr and S atoms, which are catalytically inert in isolated monolayer ZrS_2_, turn into catalytic active sites. The dramatically enhanced absorption in the entire visible light makes the ZrS_2_/B_20_ hybrid having great applications in photocatalysis or photodetection.

## Background

Atomically thin two-dimensional (2D) transition metal dichalcogenides (TMDs) have intriguing properties that make them highly suitable for many fields including lithium-ion battery, solar cell, and catalysis [1]. Thanks to the dramatic progress in recent experimental advances, many kinds of few-layer or monolayer TMDs have been successfully prepared [2, 3]. However, any one of pure-layered TMDs is not always a perfect material for different applications. To achieve superior performance for some specific cases, various strategies have been developed to engineer the chemical, physical, and electronic properties of 2D TMDs [1, 4, 5]. In particular, coupling 2D TMDs with other materials to create novel functional van der Waals (vdW) heterostructures receives growing significant attention [1].

As one of representative group IVB-TMDs, zirconium disulfide (ZrS_2_) has attracted considerable attention and shows great potential in photodetectors [6], solar cells [7], and photocatalysis [8], due to its good thermodynamic stability, environmental friendliness, high sensitivity, and low-cost production. In recent years, monolayer ZrS_2_ keeping these advantageous qualities have been successfully fabricated by various methods [9–11]. The band gap of bulk ZrS_2_ is around 1.70 eV [12, 13], while it is very interesting that mono-, bi-, and trilayer ZrS_2_ have an indirect band gap with 2.01, 1.97, and 1.94 eV [8, 14], respectively, indicating that it undergoes a transition of band gap when the dimensionality decreases from 3D to 2D. Due to its appropriate band gap, monolayer ZrS_2_ can utilize the maximum portion of the solar visible light. However, the measured efficiency in solar hydrogen production of monolayer ZrS_2_ is quite low compared with the theoretical value owing to its conduction band maximum (CBM) slightly lower than the reduction level of hydrogen [15, 16]. To overcome the drawbacks, many methods have been explored to improve the photocatalytic performance of ZrS_2_. Among them, combining with other semiconductors, such as graphene, g-C_3_N_4_, h-BN, and ZnO, has been demonstrated to be an effective strategy to enhance the stability and photocatalytic activity of ZrS_2_ [16, 17].

Boron (1s^2^ 2s^2^ 2p^2^) can form a wide variety of clusters with fascinating properties, as its neighbor carbon (1s^2^ 2s^2^ 2p^1^) which is well known showing distinct solid-state allotropes like chains, rings, and fullerenes [18]. The related study of boron clusters can date back to nearly 30 years ago [19]. A lot of boron fullerenes, such as B_80_ and B_100_ [20, 21], have been studied theoretically. Recently, Zhai et al. have firstly observed the all-boron fullerene B_40_ in experiment [22], triggering renewed interest in these boron clusters [23–25]. Herein, we for the first time study the structural and electronic properties of hybrid monolayer ZrS_2_/B_20_ vdW heterostructure to explore its potential applications in solar energy conversion by using large-scale density functional theory (DFT) computations. Here, double-ring tubular B_20_ cluster is taken as the typical boron cluster, motivated by its special structure and properties. As a stable non-planar structure formed by 20 boron atoms with high symmetry, double-ring tubular B_20_ is considered to be an important structure due to the 2D-to-3D transition of boron cluster: the boron clusters prefer 2D structures up to 19 atoms and favor 3D structures beginning at 20 atoms in terms of experimental and computational studies [26–29]. More importantly, the band gap of B_20_ ring is about 1.2~1.4 eV [19], suggesting that its spectral response covers the entire visible region, even extending to near-infrared light. It is speculated that the role of B_20_ ring in the hybrid is multiple. The calculated results show that compared to pure monolayer ZrS_2_, the ZrS_2_/B_20_ hybrid displays dramatically enhanced visible light response, making it to be great potential in solar energy conversion.

## Methods

**Fig. 1:**
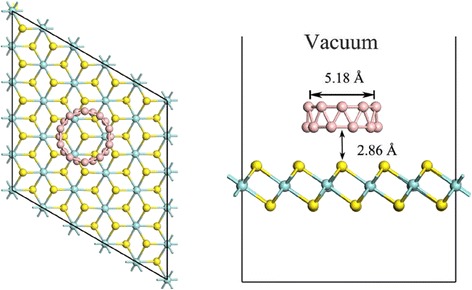
*Top* (*left*) and *side* (*right*) views of the monolayer ZrS_2_/B_20_ hybrid. *Blue-green*, *yellow*, and *pink* spheres represent Zr, S, and B atoms, respectively

The hybrid is composed of a 5 × 5 ZrS_2_ supercell and a non-planar B_20_ ring cluster, as shown in Fig. 1. A vacuum space is set to be 20 Å in order to avoid artificial interaction. All the DFT calculations are performed using CASTEP module in Materials Studio 8.0 [30]. The core electrons are described with the ultrasoft pseudopotential. The local-density approximation (LDA) with inclusion of the vdW interaction is chosen because the long-range vdW interaction is expected to be significant in such hybrid [31]. However, because the LDA functional underestimates the band gaps of semiconductors [32], all the theoretical calculations are performed using the DFT/LDA + U method. We have performed extensive tests to determine the appropriate Hubbard U parameters (Zr 4d, 3p, and S 2p are 4.0, 3.0, and 3.0 eV, respectively). The cutoff energy for the plane-wave is set to 400 eV. Geometry optimization is carried out before single point energy calculation and the force on the atoms is less than 0.03 eV/Å, the stress on the atoms is less than 0.05 GPa, the atomic displacement is less than 1.0 × 10^−3^ eV/Å, and the energy change per atom is less than 1.0 × 10^−5^ eV. For the Brillouin zone integration, a 3 × 3 × 3 Monkhorst pack *k*-point mesh is used for geometry optimization and the density-of-states (DOS) plots. The convergence of energy is 1.0 × 10^−6^ eV. To check the reliability of our results, we have also performed a test calculation with higher plane-wave cutoff energy and more *k*-points. Compared with the results given here, negligible changes are obtained for both structural and electronic structures and difference between the total energies is less than 0.03 %.

## Results and Discussion

The lattice constant of monolayer ZrS_2_ and diameter of ring B_20_ are calculated to be 3.62 and 5.18 Å, respectively, in good agreement with the previous study [14, 16, 26]. After optimization, the closest distance between a boron atom and top layer of ZrS_2_ is 2.86 Å (Fig. 1), indicating that the interaction between monolayer ZrS_2_ and B_20_ is indeed vdW rather than covalent. In order to examine the stability of the hybrid, the interface adhesion energy have been calculated, which is defined as follows:

$$ {E}_{\mathrm{ad}}={E}_{\mathrm{comb}}-{E}_{{\mathrm{ZrS}}_2}-{E}_{{\mathrm{B}}_{20}}, $$

where *E*_comb_, ${E}_{{\mathrm{ZrS}}_2}$, and ${E}_{{\mathrm{B}}_{20}}$ represent the total energy of the relaxed ZrS_2_/B_20_ hybrid, monolayer ZrS_2_, B_20_ ring, respectively. By definition, negative *E*_ad_ suggests that the adsorption is stable [33]. The interface binding energy is calculated to be −1.02 eV for this hybrid, indicating that a rather strong interaction between monolayer ZrS_2_ and B_20_ ring, and the high thermodynamically stability.

**Fig. 2:**
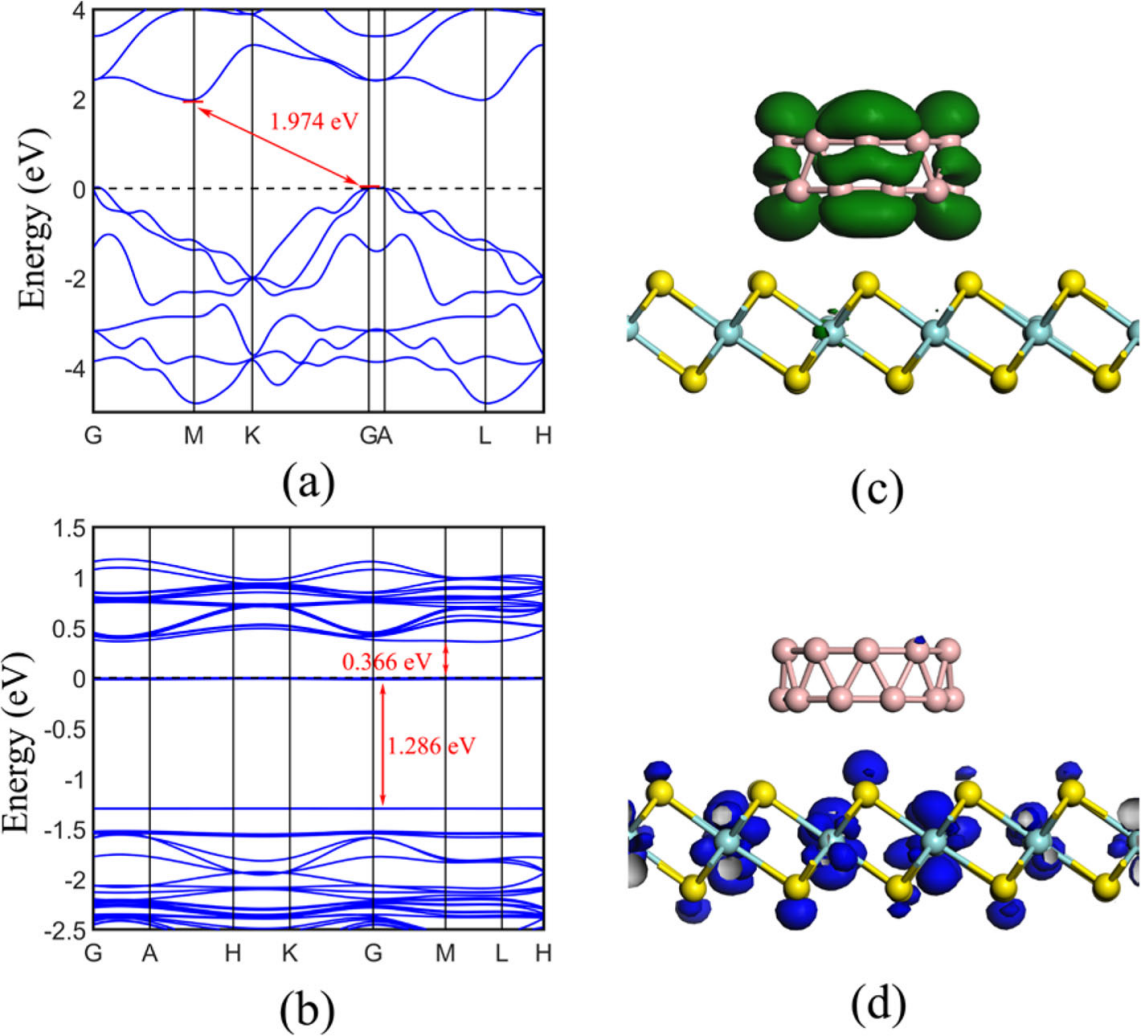
Band structures for **a** the monolayer ZrS_2_ and **b** the ZrS_2_/B_20_ hybrid. The *horizontal dashed line* indicates the Fermi level. The calculated the electron density distributions of the highest occupied (**c**) and lowest unoccupied levels (**d**) of ZrS_2_/B_20_ with an isovalue of 0.005 e/Å^3^

The band structures of monolayer ZrS_2_ and ZrS_2_/B_20_ hybrid are displayed in Fig. 2. It is obvious that monolayer ZrS_2_ has an indirect band gap of 1.97 eV, which is agree well with other results obtained from hybrid-DFT method [14, 16]. For the ZrS_2_/B_20_ hybrid, the most striking is the emergence of two almost flat bands located at about −1.3 and 0 eV, respectively (Fig. 2b). Compared to monolayer ZrS_2_, the band gap of hybrid is reduced to 0.366 eV, thus making it to be a novel material with wide spectral response, from visible light to near-infrared light.

**Fig. 3:**
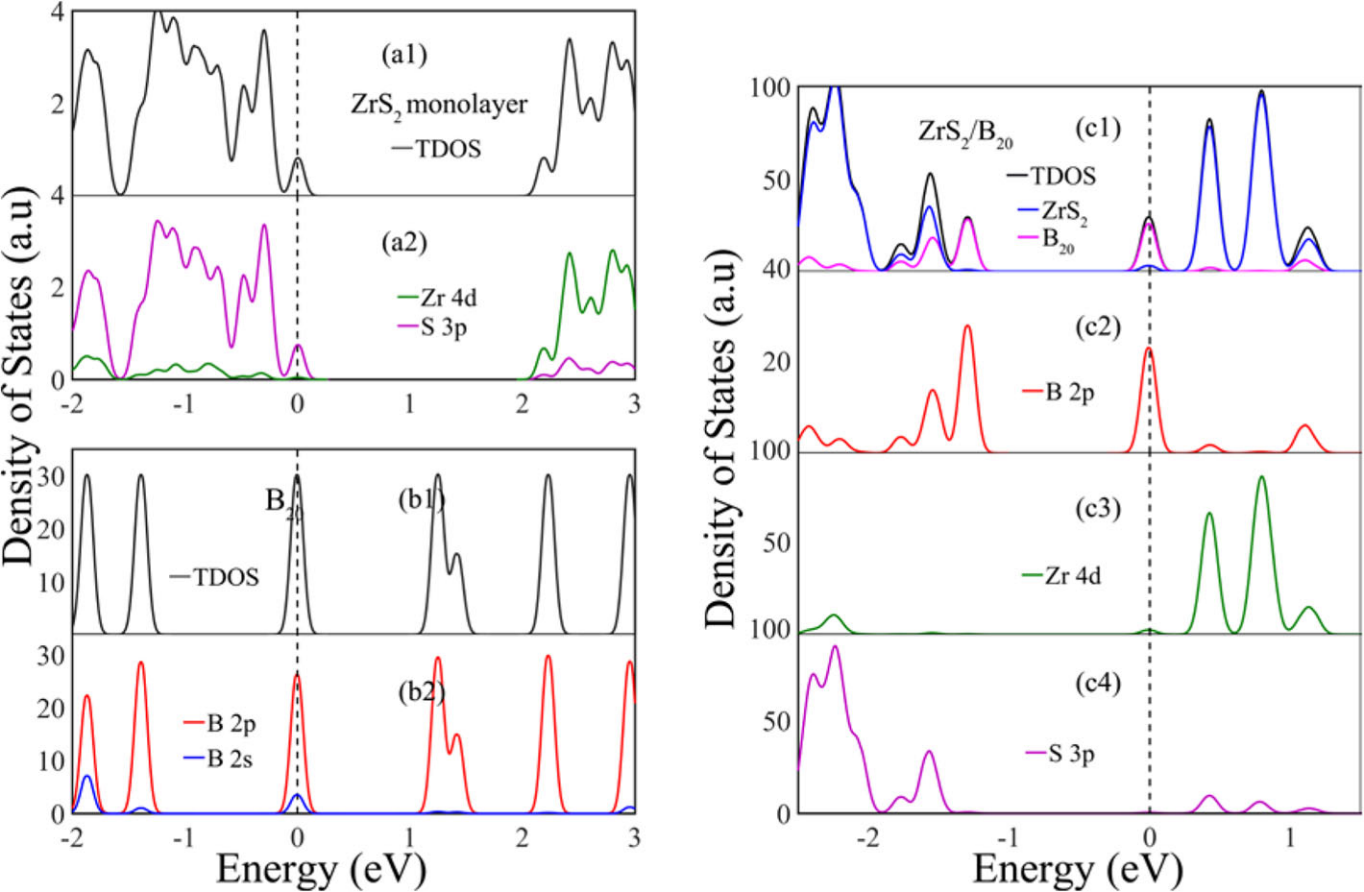
Total and partial density of states for the monolayer ZrS_2_ (*(a1)–(a2)*), ring B_20_ cluster (*(b1)–(b2)*), and the ZrS_2_/B_20_ hybrid (*(c1)–(c2)*), respectively. The *vertical dashed lines* indicate the Fermi level

To illuminate the influence of vdW interaction on the electronic properties of ZrS_2_, the total density of states (TDOS) and partial density of states (PDOS) of the monolayer ZrS_2_, double-ring tubular B_20_ cluster, and ZrS_2_/B_20_ hybrid are calculated and displayed in Fig. 3. The VB top of pure ZrS_2_ (Fig. 3 (a2)) is mainly constituted of S 3p states mixing with small Zr 4d states, while its CB bottom is composed of Zr 4d states, indicating that its near-gap electronic structure is different from that of pure MoS_2_ where the CB bottom and VB top are predominately composed of Mo 4d states [34]. Thus, pure monolayer ZrS_2_ is expected to be a better candidate than pure monolayer MoS_2_ for light absorption. For isolated ring B_20_ cluster, its VB top is mainly constituted of B 2p states mixing with small 2s states, and its CB bottom is composed of B 2p states (Fig. 3 (b2)). Obviously, this kind of near-gap electronic structure is not conducive for the electron transition of B_20_ cluster under illumination. This kind of transition-hostile near-gap electronic structure of pure ZrS_2_ or isolated ring B_20_ cluster can be changed by combining them through vdW interaction. Figure 3 (c1–c4) shows that the VB top of ZrS_2_/B_20_ hybrid is dominated by B 2p states from ring B_20_ cluster, coupled by small Zr 4d and S 3p states, whereas its CB bottom is predominately composed of Zr 4d states, which can be more clearly seen from the electron density distributions of the highest occupied and lowest unoccupied levels (HOL and LUL), respectively, as shown in Figs. 2c, d. This kind of transition-conducive near-gap electronic structure of hybrid ZrS_2_/B_20_ significantly lowers the effective band gap of the heterostructure and facilitates efficient electron-hole separation, which is the physical mechanism for high light absorption in the visible region. Note that the four nearly straight levels from −1.5 to 0 eV (Fig. 2b) are mainly composed of B 2p or S 3p states, as shown in Fig. 3. Obviously, the electron transition between these levels (i.e., B 2p or S 3p orbitals) will also significantly affect the optical properties of the ZrS_2_/B_20_ hybrid. However, owing to the electronic transition of angular momentum selection rules (*Δl* = ± 1), the transition between these levels is forbidden; thus, the electrons occupied at B 2p or S 3p states will direct transit to the CB bottom (Zr 4d orbitals), producing well-spatially separated electron-hole pairs.

**Fig. 4:**
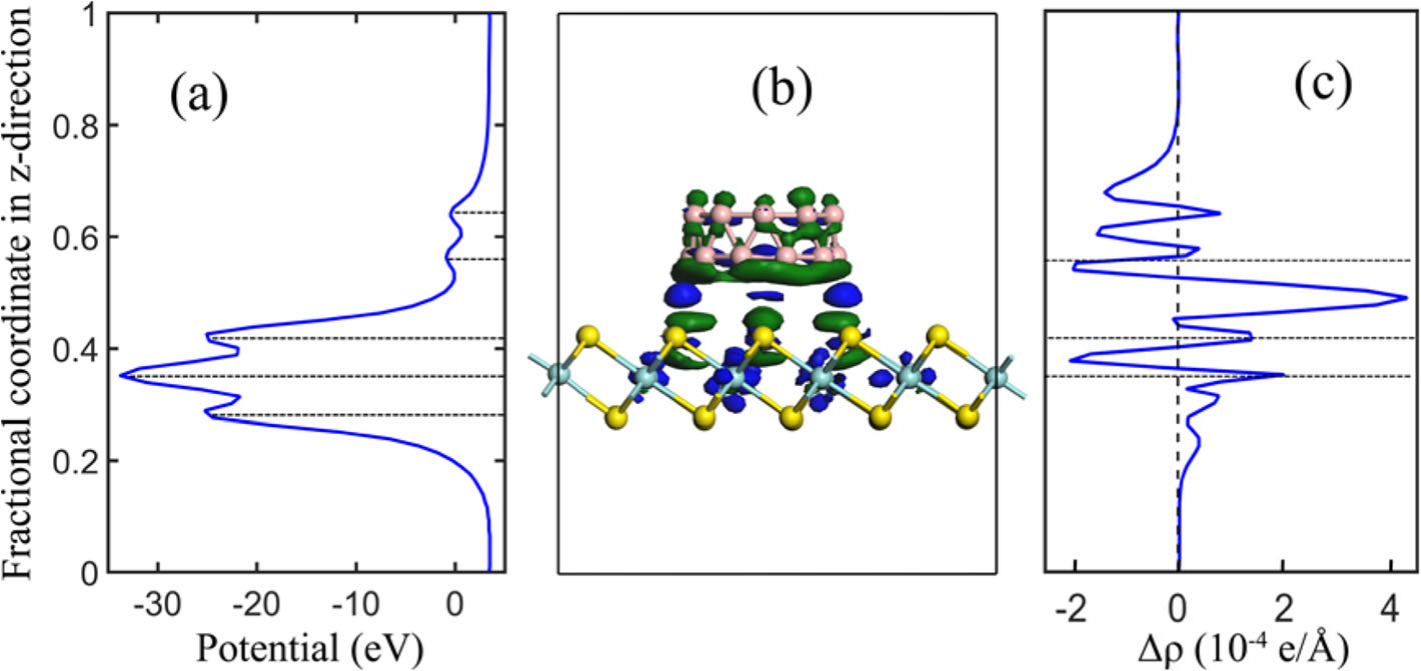
**a** Profile of the planar averaged self-consistent electrostatic potential for the ZrS_2_/B_20_ as a function of position in the z-direction. **b** 3D charge density difference for the ZrS_2_/B_20_ nanocomposite with an isovalue of 0.004 e/Å^3^. *Blue* and *green* isosurfaces represent charge accumulation and depletion in the space. **c** Profile of the planar averaged charge density difference for the ZrS_2_/B_20_ as a function of position in the z-direction. The *horizontal lines* denote the central location of each atomic layer

The variation of the DOSs implies that the interaction between ZrS_2_ and ring B_20_ cluster leads to charge transfer between the involved constituents. This can be visualized (as shown in Fig. 4b) by the three-dimensional charge density difference $\varDelta \rho ={\rho}_{{\mathrm{ZrS}}_2/{\mathrm{B}}_{20}}-{\rho}_{{\mathrm{ZrS}}_2}-{\rho}_{{\mathrm{B}}_{20}},$ where ${\rho}_{{\mathrm{ZrS}}_2/{\mathrm{B}}_{20}}$, ${\rho}_{{\mathrm{ZrS}}_2}$, and ${\rho}_{{\mathrm{B}}_{20}}$ are the charge densities of the hybrid, monolayer ZrS_2_, and ring B_20_ cluster in the same configuration, respectively. Owing to the non-covalent interaction, a very interesting charge redistribution at the hybrid ZrS_2_/B_20_ can be clearly seen, which is different from those of the MoS_2_/SnO_2_ and Ag_3_PO_4_/GR heterostructures [33, 35]. A strong charge accumulation (blue part in Fig. 4b), mainly from the B atoms at lower layer of ring B_20_ cluster and from the S atoms below the B_20_ cluster, is found just above the top S atoms. Whereas the charge depletion occurs at both sides of the S atoms below the ring B_20_ cluster and the B atoms at the cluster. Moreover, the B atoms at the lower layer (i.e., adjacent to ZrS_2_) lose more electron than those at the top layer. To offer quantitative results of charge redistribution, Fig. 4c plots the planar averaged charge density difference along the direction perpendicular to the monolayer ZrS_2_, where the positive value indicates the charge accumulation, and negative value represents the charge depletion. It is obvious that the largest efficient electron accumulation appears between the S atom and the B atom is about 4.3 × 10^−4^ e/Å^3^ and the largest efficient electron depletion occurs both at lower side of B_20_ and Zr atom are about −1.9 × 10^−4^ e/Å^3^. In order to quantitatively analyze the effective net charge variation between the two constituents, we further analyze the charge transfer by Bader method, which demonstrates that 0.302 electron transfers from B_20_ to ZrS_2_, similar to the case of the MoS_2_/C_20_ hybrid [34]. To unveil the mechanism of such an interface electron transfer in the hybrid, work functions for the ring B_20_ cluster and monolayer ZrS_2_ are calculated by aligning the Fermi level relative to the vacuum energy level. They are calculated to be 4.71 and 5.99 eV for B_20_ and monolayer ZrS_2_, respectively. The spontaneous interfacial charge transfer in the hybrid ZrS_2_/B_20_ can be simply rationalized in terms of the difference of these work functions. Most importantly, Bader analysis also shows that some Zr atoms obtains charge up to 1.768 e, while some S atoms loss charge up to 0.89 e in the ZrS_2_ layer, indicating that the vdW interaction results into some positively charged Zr atoms and negatively charged S atoms in the ZrS_2_ layer. This finding suggests that some Zr and S atoms at basal planes, initially catalytically inert, would turn out to be active sites, which would be one of the key factors to enhance the photocatalytic performance of the monolayer ZrS_2_/B_20_ hybrid.

The distribution of electric potential in the ZrS_2_/B_20_ hybrid will be altered due to the interfacial charge transfer. To display the quantitative analysis, the profile of the planar averaged self-consistent electrostatic potential for the ZrS_2_/B_20_ hybrid as a function of position in the z-direction is displayed in Fig. 4a. One can see that the electrostatic potential at two B atomic planes is lower than that at their middle region in ring B_20_ cluster, and obvious potential difference between the Zr atomic plane and two S atomic planes can be observed, rendering a typical S-Zr-S sandwich distribution. Note that the potential at the upper S atomic plane is slightly higher than that at the lower S atomic plane (upper −25.05 eV, lower −25.26 eV), verifying that the S atoms at the upper layer lose some electrons due to the ring B_20_ cluster modification (as displayed in Fig. 4b). The potential at the monolayer ZrS_2_ plane is much lower than that at ring B_20_ cluster, resulting into a large potential difference between the two constituents. The built-in potential at the interface promotes the separation of electron-hole pairs. Moreover, under light irradiation, the separation and migration of photogenerated carriers at the interface will be more effective due to the appearance of this built-in potential, i.e., the existence of a potential well can effectively hinder the recombination of photogenerated charge carriers in the ZrS_2_/B_20_ hybrid. The results suggest that the ZrS_2_/B_20_ hybrid would be a potential photocatalyst with high quantum efficiency.

**Fig. 5:**
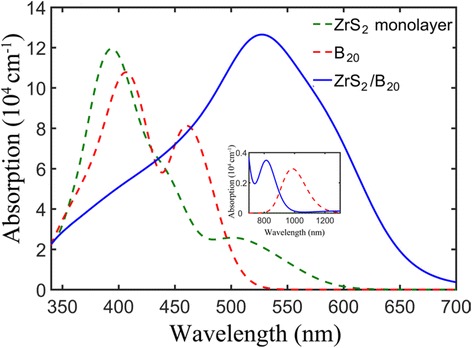
Calculated absorption spectra of the monolayer ZrS_2_ (*green dashed line*), ring B_20_ cluster (*red dashed line*), and the ZrS_2_/B_20_ hybrid (*blue solid line*) for the polarization vector perpendicular to the surface. *Inset*: the absorption spectra from 700 to 1300 nm

The high light harvesting is vital for a high-efficiency photocatalyst or photodetector except for a low recombination rate of photogenerated carriers. It has been demonstrated that non-covalent modification by graphene or monolayer MoS_2_ can extend the absorption edge of semiconductors (like TiO_2_, AgPO_4_ and SnO_2_) to the vis-light region [33, 35, 36]. Similarly, coupling fullerene with photocatalysts is also an effective strategy to enhance the light absorption [34]. To explore the influence of ring B_20_ cluster on the light absorption of ZrS_2_, the UV-vis absorption spectra of the ZrS_2_/B_20_ hybrid, and its constituents are calculated, as shown in Fig. 5. For monolayer ZrS_2_, the optical absorption edge occurs at about 620 nm, which is attributed to the intrinsic transition from the S 3d to Zr 4d orbital, in agreement with other theoretical results [16]. This adsorption edge. Owing to its small band gap (as shown in Fig. 3b), the isolated ring B_20_ cluster can absorb some near-infrared light (800~1200 nm), but the absorb intensity is very weak, as shown in Fig. 5. Part of visible light (<520 nm) can also be absorbed by the isolated ring B_20_ cluster. The most striking feature in Fig. 5 is that visible light response of the ZrS_2_/B_20_ hybrid has been dramatically enhanced in the region from 450 to 700 nm compared to that of the monolayer ZrS_2_. That is to say, the ZrS_2_/B_20_ hybrid very efficiently absorb most of the visible light. The significant increase of optical absorption of the ZrS_2_/B_20_ hybrid is close related to the unique near band-gap electronic structure (Fig. 3). Considering that the separation and migration of photogenerated carriers in the hybrid will be facilitated due to the existence of the built-in potential at the interface, one can conclude that the ZrS_2_/B_20_ hybrid would be an active photocatalyst or photodetector in the main part of the solar spectrum, and ever poor illumination of interior lighting.

## Conclusions

In summary, we have studied the electronic structure, charge transfer, and optical properties of the ZrS_2_/B_20_ hybrid by using DFT calculation. It is found that the band gap and near-gap electronic structure of the monolayer ZrS_2_ can be tuned by the non-covalent modification of double-ring tubular B_20_ cluster. The interfacial charge transfer results into some positively charged Zr atoms and negatively charged S atoms in the hybrid, thus to be active sites, which are initially catalytically inert in the isolated monolayer ZrS_2_. The ZrS_2_/B_20_ hybrid exhibits dramatically enhanced absorption in the entire visible light due to its small band gap and unique near-gap electronic structure caused by interfacial interaction. These results suggest that not only the ZrS_2_/B_20_ hybrid would be an active photocatalyst or photodetector in the main part of the solar spectrum, and ever poor illumination of interior lighting, but also ring B_20_ cluster modification would be an effective strategy to tune the performance of monolayer TMDs.
